# AICAR, an AMP-Activated Protein Kinase Activator, Ameliorates Acute Pancreatitis-Associated Liver Injury Partially Through Nrf2-Mediated Antioxidant Effects and Inhibition of NLRP3 Inflammasome Activation

**DOI:** 10.3389/fphar.2021.724514

**Published:** 2021-08-31

**Authors:** Lijun Kong, Hewei Zhang, Chaosheng Lu, Keqing Shi, Hongjian Huang, Yushu Zheng, Yongqiang Wang, Dan Wang, Hongwei Wang, Wei Huang

**Affiliations:** ^1^ Key Laboratory of Diagnosis and Treatment of Severe Hepato-Pancreatic Diseases of Zhejiang Province, The First Affiliated Hospital of Wenzhou Medical University, Wenzhou, China; ^2^ Department of Pediatrics, The First Affiliated Hospital of Wenzhou Medical University, Wenzhou, China; ^3^ Translational Medicine Laboratory, The First Affiliated Hospital of Wenzhou Medical University, Wenzhou, China; ^4^ Department of Nutrition, The First Affiliated Hospital of Wenzhou Medical University, Wenzhou, China

**Keywords:** pancreatitis, liver injury, AMPK, Nrf2, NLRP3 inflammasome

## Abstract

Acute pancreatitis (AP) is a highly fatal acute inflammation and is often accompanied by multiple organ dysfunction syndrome (MODS). The liver, one of the most vulnerable extrapancreatic organs in AP, is the major organ involved in the evolution of the disease and correlates strongly with the occurrence of MODS. However, the etiology of pancreatitis-associated liver injury (PALI) has not been clarified and currently lacks an effective treatment. 5-Aminoimidazole-4-carboxamide ribonucleotide (AICAR) is a cell permeable nucleoside with pleiotropic effects on anti-inflammatory and antioxidant stress that binds with adenosine monophosphate protein kinase (AMPK) and induces AMPK activation. However, the role of AICAR in PALI remains elusive. Here, we show that activation of AMPK by AICAR, a direct AMPK agonist, significantly ameliorates sodium taurocholate-induced PALI in rats, whereas treatment of PALI rats with the AMPK antagonist Compound C profoundly exacerbates the degree of liver injury, suggesting that hepatic AMPK activation exerts an essential protective role in PALI. Mechanistically, AICAR induces AMPK activation, which in turn activates nuclear factor erythroid 2-related factor 2(Nrf2) -regulated hepatic antioxidant capacity and inhibits NLRP3 inflammasome-mediated pyrolysis, protecting rats from sodium taurocholate-induced PALI. In addition, Nrf2 deficiency strikingly weakens the beneficial effects of AICAR on alleviation of liver injury, oxidative stress and NLRP3 inflammasome activation in L-arginine-induced PALI mice. Thus, AICAR protects against PALI in rodents by triggering AMPK, which is mediated at least in part by Nrf2-modulated antioxidant effects and NLRP3 inflammasome activation.

## Introduction

Acute pancreatitis (AP) is a highly fatal acute inflammation with rapid progression. (Argaiz and de Moraes, 2021). There is an increasing prevalence of AP, with an estimated 33.74 cases per 100,000 people worldwide each year (Garg and Singh, 2019). The high mortality of AP is largely attributed to multiple organ dysfunction syndrome (MODS), such as liver or lung injury (Shi et al., 2020). The liver, a major organ involved in the evolution of AP, is strikingly vulnerable to inflammatory cytokines and correlates strongly with the occurrence of MODS (Li S. et al., 2019; Mei et al., 2019). Pancreatitis-associated liver injury (PALI) is a serious and even fatal complication in the development of AP (Wang et al., 2018). Mechanistically, a series of endogenous vasoactive substances released during AP lead to hepatic microcirculation disturbance, which is the essential cause of liver injury (Wenhong et al., 2012). Liver injury in turn triggers a systemic inflammatory response by affecting toxin metabolism and releasing massive inflammatory mediators (Rios et al., 2010; Ou et al., 2017). The PALI phenomenon has been observed in both clinical and experimental settings (Wang et al., 2006; Wenhong et al., 2012; Lv et al., 2015; Bakır et al., 2016; Ou et al., 2017). Notably, Curbey et al. reported that 80% of AP patients had liver damage and the liver injury aggravates the degree of pancreatitis if it is not intervened in time, which significantly prolongs the course of pancreatitis (Ou et al., 2017; Li X. et al., 2019). To date, the etiology of PALI has not been clarified, and no effective treatment has been developed. Thus, finding a promising therapeutic way to prevent or treat this devastating and fatal disease is urgently needed.

Adenosine monophosphate protein kinase (AMPK) is the main energy receptor regulating cell metabolism (Yang et al., 2021; Zhao et al., 2021). The activation of AMPK depends on the phosphorylation of Thr172 of the α subunit (Garcia and Shaw, 2017). In general, AMPK is activated under energy stress by sensing an increase in the ratios of AMP/ATP and ADP/ATP, thereby stimulating the catabolic process and maintaining energy homeostasis (Lin and Hardie, 2018). In addition to its role in energy dynamic balance, activated AMPK is also associated with reducing redox stress, inhibiting inflammation or limiting cell proliferation (Zimmermann et al., 2015; Liu et al., 2020; Chin et al., 2021). Recent studies indicate that AMPK activation plays an inhibitory role in mediating ethanol-induced oxidative stress in human pancreatic acinar cells, whereas AMPK inhibition can aggravate liver inflammation in mice with hepatic ischemia-reperfusion (Zhou et al., 2018; Srinivasan et al., 2020). Although the anti-inflammatory and antioxidant stress functions of AMPK activation have been well characterized, little is known about the pathophysiological roles in PALI, and the potential mechanism whereby AMPK activation protects against PALI remains poorly defined.

Under physiological conditions, serine 374, 408 and 433 of nuclear factor erythroid related factor 2 (Nrf2) are hyperphosphorylated with the activation of AMPK (Matzinger et al., 2020). Early studies have reported that AMPK activation enhances cellular antioxidant potential by activating the transcriptional activity of Nrf2 (Garcia and Shaw, 2017; Xu et al., 2020). Nrf2 is a member of the Cap’n’Collar (CNC) family of basic leucine zipper (bZIP) transcription factors, which activates the endogenous antioxidant defense system through translocation into the nucleus, thus combating inflammation and oxidative stress (Tonelli et al., 2018; Lyu et al., 2020; Gong et al., 2021; Tan et al., 2021). Inflammation causes oxidative stress; in turn, oxidative stress also boosts the production of inflammatory cytokines through activation of proinflammatory pathways, including the known Nod-like Receptor Protein 3 (NLRP3) inflammasome pathway (Zeng et al., 2017). It consists of NLRP3 scaffold, adaptor protein apoptosis-associated speck-like protein (ASC) and proinflammatory caspase-1 (Zou et al., 2021). When NLRP3 is activated, the p20 subunit and p10 subunit are heterodimerized to form bioactive caspase-1, which processes the release of IL-1β and induces a unique inflammatory type of cell death called pyroptosis (Chen et al., 2017; Unamuno et al., 2021). Growing evidence has established that NLRP3 inflammasome activation leads to hepatocyte pyroptosis and severe liver inflammation (Han et al., 2018; Wree et al., 2018). Recently, the inverse correlation between the activated Nrf2 and NLRP3 inflammasome pathway that inhibits inflammation through augmenting antioxidant capacity in mice have been proved in acute lung injury (Liu et al., 2017). Nonetheless, whether the activation of AMPK plays an antioxidative and anti-inflammatory role through Nrf2-mediated antioxidant effects and inhibition of the NLRP3 inflammasome pathway in PALI remain to be determined.

5-Aminoimidazole-4-formamide ribonucleotide (AICAR) is a kind of cellular permeable nucleoside that activates AMPK to play anti-inflammatory and antioxidant stress effects (Swinnen et al., 2005; Bone et al., 2017; Kaphalia et al., 2019). AICAR is converted into ZMP (an AMP mimic) in cells to bind to the γ subunit of AMPK, causing conformational changes in the enzyme and promoting the phosphorylation of Thr172 of the AMPKα subunit by liver kinase B1 (LKB1) or other upstream AMPK kinases (AMPKK) (Sun et al., 2007; Hill et al., 2016; Lane et al., 2020; Višnjić et al., 2021). Emerging evidence indicates that the activation of AMPK by AICAR attenuates high glucose-induced oxidative stress in rat cardiomyocytes (Shen et al., 2019). Moreover, the direct AMPK agonist AICAR negatively regulates the IL-6-stimulated inflammatory response in human liver cells by suppressing the phosphorylation of STAT3 (Nerstedt et al., 2010). However, it is not clear whether pharmacological activation of AMPK by the direct AMPK small molecule agonist AICAR is a therapeutic strategy for PALI.

In the present study, we investigated whether activation of AMPK by AICAR limits the inflammatory response and oxidative stress in the progression of PALI in two rodent models of severe acute pancreatitis (SAP) *via* Nrf2-mediated antioxidant effects and NLRP3 inflammasome activation. Our results provide the first direct evidence of the beneficial effects of pharmacological activation of AMPK by AICAR against the progression of PALI, including reduced redox stress and decreased NLRP3 inflammasome activation. Moreover, Nrf2 deficiency dramatically weakened these beneficial effects of AICAR in L-arginine-induced PALI mice. Thus, AICAR protects against PALI at least in part through Nrf2-mediated antioxidant effects and inhibition of NLRP3 inflammasome activation.

## Materials and Methods

### Animal Experiments

Male SD rats (220–250 g, age 7–8 weeks) were obtained from the Experimental Animal Center of Wenzhou Medical University. Nrf2-knockout (Nrf2^−/−^) mice on C57BL/6 background were procured from Jackson Laboratory (Bar Harbor, Maine, United States). These homozygous Nrf2^−/−^ mice were backcrossed with C57BL/6 wild type mice. Heterozygous offspring were then further bred to gain wild type and Nrf2^−/−^ littermates. Genotypes of Nrf2^−/−^ mice were identified by PCR. All rats and mice were fed randomly at 24 ± 2°C and 40–60% humidity with a 12 h dark cycle before the experiment. All animal procedures were reviewed and approved by the Animal Ethics Committee of Wenzhou Medical University.

Wild-type rats were randomly divided into four groups: the control group (n = 10), severe acute pancreatitis (SAP) group (n = 10), AMPK agonist (AICAR) group (n = 8) and AMPK inhibitor (Compound C, CC) group (n = 8). Sham operation was performed in the control group. Pancreatitis was induced in the SAP, AICAR and CC groups by retrograde injection of 3% sodium taurocholate (STC, 0.1 ml/100 g of body weight, YZ-110815, Solarbio, Beijing, China) *via* the pancreatic duct using a syringe pump as previously described (Fang et al., 2020). The AICAR group received intraperitoneal injection of AICAR (400 mg/kg) 1 h before the operation. In the CC group, CC (13.8 mg/kg) was also injected intraperitoneally 1 h before the operation. AMPK agonist (AICAR) and AMPK inhibitor (CC) were purchased from MedChemExpress (HY-13417, HY-13418A, MedChemExpress, New Jersey, United States). The specific dosages of AICAR and CC used in this study based in the description in previous studies with minor modifications (Saito et al., 2012; Guo et al., 2014; Martin et al., 2019). 24 h after injection of sodium taurocholate, the rats were anesthetized with isoflurane (in 4% for induction and 3% for maintenance; R510, RWD Life Science, Shenzhen, China) on the anesthetic machine. Rats were sacrificed, and pancreatic tissues, liver tissues and blood samples were collected for further study.

Male C57BL/6 mice or Nrf2^−/−^ mice were randomly divided into three groups: the control group (n = 6), SAP group (n = 6) and AMPK agonist (AICAR) group (n = 6). The control group mice were intraperitoneally injected with 0.9% normal saline. In the SAP and AICAR groups, the pancreatitis model was established by intraperitoneal injection of 8% L-arginine (pH = 7.0, 4.0 g/kg, A5006, Sigma, Missouri, United States) twice at an interval of 1 h as previously described (Liu et al., 2016). In the AICAR group, mice were intraperitoneally injected with AICAR (400 mg/kg) twice: 1 h before and 24 h after model establishment. 48 h after the injection of L-arginine, the mice were anesthetized with isoflurane (in 3.5% for induction and 2.5% for maintenance) on the anesthesia machine. Finally, the mice were sacrificed, and pancreatic tissues, liver tissues and blood samples were collected for subsequent experiments.

### Histopathological Analysis

Pancreatic and liver tissues were collected, fixed immediately in 4% paraformaldehyde for 24  h, dehydrated in a graded ethanol series, and then embedded in paraffin. The tissue blocks were cut into 4.5 μm-thick sections, dewaxed, and hydrated. Then, pancreatic and liver sections were stained with hematoxylin and eosin (H&E) staining (G1120, Solarbio, Beijing, China) according to the manufacturer’s instructions. After observation under an Olympus BX-51 microscope (Olympus Corporation, Tokyo, Japan), histological scores were obtained to evaluate the degree of pancreatic and liver injury with Image-Pro Plus 6.0 software (Media Cybernetics, Bethesda, United States) as described elsewhere (Rongione et al., 1997; Elshal et al., 2019).

### Immunohistochemistry Analysis

Immunohistochemistry was used to qualitatively analyze the expression of phenotypic markers. The liver sections (4.5 μm) were boiled in antigen retrieval buffer containing citrate-hydrochloric acid (C8532, Sigma, Missouri, United States) for 15 min. Then, hydrogen peroxide (3%) was used for 10 min to block the activity of endogenous peroxidase and subsequently blocked with 5% bovine serum albumin (A1933, Sigma, Missouri, United States) for 30 min at 37°C. Primary antibodies for IL-6 (1:200; MB9296, Bioworld Technology, Minnesota, United States), IL-1β (1:200; BS6067, Bioworld Technology, Minnesota, United States), TNF-α (1:200; BS6000, Bioworld Technology, Minnesota, United States), MCP-1 (1:200; DF7577, Affinity, Ohio, United States), HO-1 (1:200; ab13243, Abcam, Massachusetts, United States) and NQO-1 (1:200; ab28947, Abcam, Massachusetts, United States) were added and incubated at 4°C overnight. After washing, the sections were incubated with secondary antibodies (1:200; A0277, Beyotime Institute of Biotechology; Goat anti-rabbit IgG-HRP) for 1 h at 37 °C. Finally, the slides were stained using diaminobenzidine (DAB, P0202, Beyotime, Shanghai, China) for color visualization. Images of representative tissue spots were captured with an Olympus BX-51 microscope (Olympus Corporation, Tokyo, Japan) and analyzed with Image-Pro Plus 6.0 software (Media Cybernetics, Bethesda, United States).

### Biochemical Indexes Assay

Fresh pancreatic and liver tissues and blood samples were collected for biochemical analysis. The levels of serum amylase and lipase were measured by assay kit (C016-1-1, A054-1-1, Nanjing Jiancheng Bioengineering Institute, Nanjing, China) to evaluate the degree of pancreatitis. The serum levels of alanine aminotransferase (ALT) and aspartate aminotransferase (AST) were measured with commercial kit (C009-2-1, C010-2-1, Nanjing Jiancheng Bioengineering Institute, Nanjing, China) to evaluate the degree of liver injury and function. The contents of malondialdehyde (MDA) and superoxide dismutase (SOD) in pancreas and liver homogenate were determined with commercial kit (A003-1-2, A001-3-2, Nanjing Jiancheng Bioengineering Institute, Nanjing, China). The contents of myeloperoxidase (MPO) were measured with commercial kit (A044-1-1, Nanjing Jiancheng Bioengineering Institute, Nanjing, China). All measurements were conducted according to the manufacturer’s instructions of the assay kit.

### Western Blot Analysis

The liver tissues were homogenized in RIPA lysis buffer (P0013C, Beyotime, Shanghai, China), and then the extract was transferred to a centrifuge tube and centrifuged at 12,000 rpm for 20 min. After testing the total protein concentration with a BCA protein analysis kit (P0012S, Beyotime, Shanghai, China), 40 μg of protein sample was separated on a 10% sodium dodecyl sulfate-polyacrylamide gel and transferred to a PVDF membrane. The membrane was blocked with 5% skim milk for 1 h and then incubated with specific primary antibodies against *p*-AMPK (Thr172; 1:1,000; 8208S, Cell Singling Technology, Boston, United States), AMPK (1:1,000; 5832S, Cell Singling Technology, Boston, United States), CD68 (1:1,000; ab125212, Abcam, Massachusetts, United States), NLRP3 (1:1,000; ab263899, Abcam, Massachusetts, United States), Caspase-1 (1:1,000; 22915-1-AP, Proteintech, Wuhan, China), cleaved IL-1β (1:1,000; AF4006, Proteintech, Wuhan, China), Nrf2 (1:1,000; 20733S, Cell Singling Technology, Boston, United States), HO-1 (1:1,000; ab13243, Abcam, Massachusetts, United States), NQO-1 (1:1,000; ab28947, Abcam, Massachusetts, United States), Lamin B (1:1,000; 12987-1-AP, Proteintech, Wuhan, China) and GAPDH (1:5,000; A00227-1, Boster, Wuhan, China) at 4°C overnight. All membranes were washed with TBST 3 times and incubated with horseradish peroxidase-conjugated secondary antibody (1: 5,000; A25012, Abbkine Scientific Co., Ltd, Wuhan, China) for 1 h. Finally, the bands were visualized using enhanced chemiluminescence (WP20005, Thermo Fisher Scientific, California, United States), and densitometry analysis was performed using VisionWorks imaging software (Eastman Kodak Company, New York, United States).

### Nuclear Protein Extraction

Nuclear components were extracted from fresh liver tissues using a nuclear protein extraction kit (P0027, Beyotime, Shanghai, China) according to the manufacturer’s instructions.

### RNA Extraction and qRT-PCR

Total RNA was extracted from liver tissues with TRIzol Reagent (15596026, Thermo Fisher Scientific, California, United States) according to the manufacturer’s specifications. Reverse transcription was completed by a Prime-Script RT Master Mix transcription kit (RR036A, TaKaRa, Tokyo, Japan). mRNA-specific primers (Sangon Biotech, China) were used to detect the RNA expression of IL-6, IL-1β and TNF-α. A 7500 Fast system (Applied Biosystems, United States) was used for qRT-PCR analysis. The results were analyzed using the 2^−ΔΔCt^ method, and GAPDH was amplified as an internal standard. The primer sequences are listed in Supplementary Table S1.

### Statistical Analysis

All experiments were independently repeated three times. Values are presented as the mean ± SD (standard deviation). Statistical analysis was performed using GraphPad Prism 7.0 software (GraphPad Software, Inc., La Jolla, CA United States). Statistical significance was assessed by Student’s t-test and one-way analysis of variance. Multiple comparisons between groups were analyzed using Tukey’s post hoc test. *p* < 0.05 was considered statistically significant.

## Results

### Direct AMPK Agonist AICAR Attenuates Pancreatitis-Associated Liver Injury and Restores Liver Function in Sodium Taurocholate-Induced SAP Rats

**FIGURE 1:**
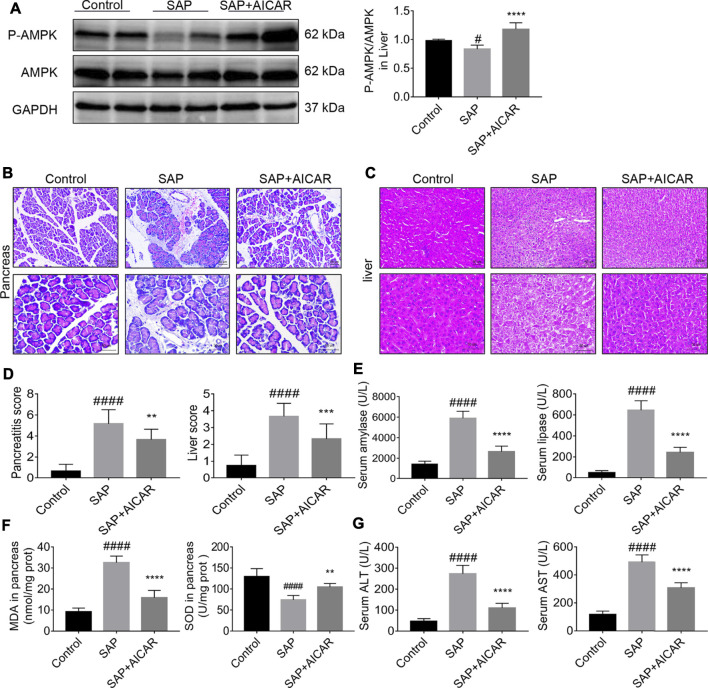
The therapeutic effects of AICAR on pancreatitis-associated liver injury (PALI) in sodium taurocholate-induced SAP rats. 7–8 week-old wild-type male SD rats were randomly divided into three groups: the control group (control, n = 10) was treated with sham operation and injected with 0.9% normal saline; the severe acute pancreatitis (SAP) group (n = 10) was infused with 3% sodium taurocholate (0.1 ml/100 g); and the AMPK agonist (AICAR) group (n = 8) received intraperitoneal injection of AICAR (400 mg/kg) 1 h before the operation. **(A)** The protein expression levels of phosphorylated AMPK (*p*-AMPK) and AMPK were detected by Western blot, and the relative ratios of *p*-AMPK/AMPK were quantified by VisionWorks imaging software. The normalized values are indicated in the histogram. GAPDH expression was used as loading control. **(B,C)** Representative pathological images of the pancreas and liver (200x and 400x) under a light microscope (H&E staining, Bar = 50 μm). **(D)** Histological scores were obtained to evaluate the degree of pancreas and liver injury from H&E staining. **(E)** Serum amylase and lipase levels in rats. **(F)** The MDA and SOD contents in pancreatic tissues of rats in each group. **(G)** Detection of serum ALT and AST activity. Data are presented as the mean ± SD of three independent experiments. ^#^ *p* < 0.05, ^##^ *p* < 0.01, ^###^ *p* < 0.001, ^####^ *p* < 0.0001 vs. control group; ^*^ *p* < 0.05, **p < 0.01, ***p < 0.001, ****p < 0.0001 vs. SAP group.

We investigated whether the direct AMPK activator AICAR (400 mg/kg) alleviates pancreatitis-associated liver injury by AMPK activation in a rat model of sodium taurocholate-induced SAP. We first validated the AMPK activator activity of AICAR in liver tissues of sodium taurocholate-induced SAP rats. Interestingly, the ratio of *p*-AMPK/AMPK in liver tissues significantly decreased after sodium taurocholate treatment. However, administration of AICAR by intraperitoneal injection into sodium taurocholate-treated rats resulted in a dramatic elevation of this ratio, suggesting that the reduced AMPK phosphorylation levels in the liver tissues of SAP rats were effectively activated by AICAR (Figure 1A). Hematoxylin and eosin staining demonstrated that the pancreas in SAP rats presented with notable interstitial edema, inflammatory cell infiltration and hemorrhagic necrosis, with the pancreatitis score analysis indicating a >5-fold increase over the control (Figures 1B,D). Likewise, the liver tissues of SAP rats were accompanied by obvious hepatocyte edema, necrosis, liver sinusoid congestion and dramatically damaged hepatic lobular structure, with the liver score analysis indicating a >4-fold increase compared with the control groups (Figures 1C,D). However, these negative changes were markedly reduced in the AICAR treatment groups (Figures 1B–D). Consistent with our histologic findings, the plasma levels of amylase and lipase, two common clinical indicators for assessing pancreatic injury (Steinberg et al., 2017), were elevated in SAP rats compared with control rats, whereas AICAR treatment prevented these increases (Figure 1E). Meanwhile, the increased levels of MDA and decreased concentrations of SOD in the pancreas of SAP rats indicated oxidative stress injury and downregulated antioxidant ability of the pancreas; however, administration of AICAR in rats significantly decreased the levels of MDA and increased the concentrations of SOD in pancreatic tissues of SAP rats, suggesting that AICAR supplementation restores the antioxidant ability of the pancreas in sodium taurocholate-induced SAP rats (Figure 1F). Correspondingly, evaluation of liver function in the SAP rats indicated that both the serum values of ALT and AST were significantly increased. In the presence of the AMPK activator AICAR, liver function was improved (Figure 1G). Taken together, the above results suggest that sodium taurocholate infusion established a successful rat model of PALI and that supplementation with AICAR not only reduces the severity of pancreatitis but also attenuates PALI and restores liver function in sodium taurocholate-induced SAP rats.

### AICAR Prevents SAP-Induced Hepatic Inflammation in a Sodium Taurocholate-Induced SAP Rat Model

**FIGURE 2:**
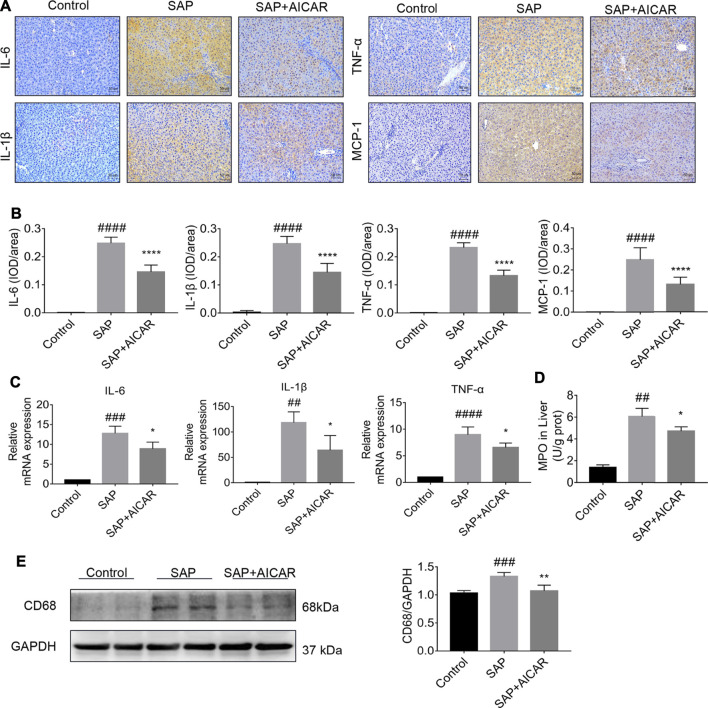
AICAR protects against SAP-induced hepatic inflammation in a sodium taurocholate-induced PALI rat model. 7–8 week-old wild-type male SD rats were randomly divided into three groups: the control group (control, n = 10) was treated with sham operation and injected with 0.9% normal saline; the severe acute pancreatitis (SAP) group (n = 10) was infused with 3% sodium taurocholate (0.1 ml/100 g); and the AMPK agonist (AICAR) group (n = 8) received intraperitoneal injection of AICAR (400 mg/kg) 1 h before the operation. **(A,B)** The protein expression of IL-6, IL-1β, TNF-α and MCP-1 in liver sections was detected by immunohistochemistry (original magnification ×200, Bar = 50 μm). Image-Pro Plus 6.0 software was used for statistical analysis. **(C)** The mRNA levels of IL-6, IL-1β, and TNF-α in liver tissues were measured by RT-qPCR. **(D)** MPO contents in liver tissues. **(E)** The protein expression of CD68 was assessed by Western blot and quantified by densitometry using VisionWorks imaging software. GAPDH expression was used as a loading control. Each value represents the mean ± SD. ^#^ *p* < 0.05, ^##^ *p* < 0.01, ^###^ *p* < 0.001, ^####^ *p* < 0.0001 vs control group; ^*^ *p* < 0.05, **p < 0.01, ***p < 0.001, ****p < 0.0001 vs. SAP group.

We explored the hypothesis that the molecular basis of AICAR in improving PALI is attributed to its anti-inflammatory capability. Our results indicated that the SAP rats exhibited higher hepatic expression of monocyte chemotactic protein 1 (MCP-1, a protein that specifically affects chemotaxis and activates macrophages) and CD68 (a rat macrophage marker) as well as increased concentrations of MPO (a functional and activation marker of neutrophils) in liver tissues compared with the control groups, whereas AICAR treatment profoundly downregulated the expression levels of these inflammatory mediators (Figures 2A,B,D,E). In addition, immunohistochemical staining and qRT-PCR indicated that the protein and messenger RNA (mRNA) expression levels of IL-6, IL-1β and TNF-α were significantly elevated in the liver tissues of SAP rats compared with the control group; however, replenishment of AICAR inhibited the increased expression of these inflammatory cytokines (Figures 2A–C). These observations confirm that AICAR treatment protects against PALI in sodium taurocholate-induced SAP rats, likely by inhibiting the inflammatory response in the liver.

### AICAR Treatment Markedly Increases the Antioxidant Abilities of the Liver in Sodium Taurocholate-Induced SAP Rats

**FIGURE 3:**
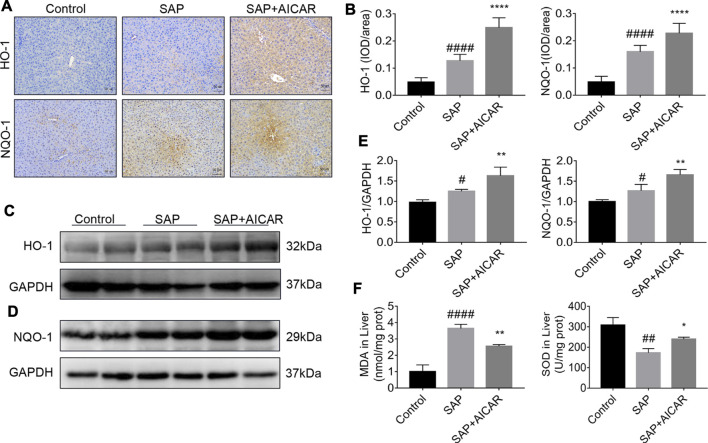
AICAR prevents hepatic oxidative stress in a sodium taurocholate-induced PALI rat model. 7–8 week-old wild-type male SD rats were randomly divided into three groups: the control group (control, n = 10) was treated with sham operation and injected with 0.9% normal saline; the severe acute pancreatitis (SAP) group (n = 10) was infused with 3% sodium taurocholate (0.1 ml/100 g); and the AMPK agonist (AICAR) group (n = 8) received intraperitoneal injection of AICAR (400 mg/kg) 1 h before the operation. **(A,B)** Liver sections were collected and stained with antibodies against HO-1 and NQO-1, and the average integrated optical density (IOD)/area of HO-1 and NQO-1 was quantified using Image-Pro Plus 6.0 software (original magnification ×200, Bar = 50 μm). **(C–E)** The protein expression levels of HO-1 and NQO-1 in liver tissues were assessed by Western blot and quantified by densitometry using VisionWorks imaging software. The level of GAPDH was used as a loading control. **(F)** MDA and SOD contents were measured in liver tissues. Data are presented as the mean ± SD obtained from three independent experiments. ^#^ *p* < 0.05, ^##^ *p* < 0.01, ^###^ *p* < 0.001, ^####^ *p* < 0.0001 vs control group; ^*^ *p* < 0.05, **p < 0.01, ***p < 0.001, ****p < 0.0001 vs. SAP group.

To further determine the roles of AICAR in PALI, we next investigated whether replenishment of AICAR can rescue the damaged antioxidant system in sodium taurocholate-induced SAP rats. Not surprisingly, immunohistochemical staining and Western blot results indicated that the hepatic expression levels of antioxidant proteins heme oxygenase-1 (HO-1) and NADPH quinone oxidoreductase 1 (NQO-1) were slightly upregulated in sodium taurocholate-induced SAP rats, which may represent a stress protection for the liver to defend against pancreatitis-induced liver injury. Notably, AICAR supplementation further augmented the hepatic expression levels of HO-1 and NQO-1 after sodium taurocholate treatment in rats (Figures 3A–E). Furthermore, the detection results of hepatic tissues in sodium taurocholate-induced SAP rats showed that the levels of MDA were significantly elevated, whereas the concentrations of SOD were strikingly decreased, suggesting that the antioxidant capacity of the liver in sodium taurocholate-induced SAP rats was disrupted. However, treatment with AICAR significantly restored the antioxidant abilities of the liver, as evidenced by an obvious elevation in hepatic concentrations of SOD and a marked decline in the hepatic levels of MDA (Figure 3F). These data suggest that AICAR supplementation prevents sodium taurocholate-induced PALI in rats by increasing antioxidant activities in the liver.

### AICAR Treatment Activates the AMPK/Nrf2 Signaling Pathway and Inhibits NLRP3 Inflammasome Activation in the Liver Tissues of Sodium Taurocholate-Induced SAP Rats

**FIGURE 4:**
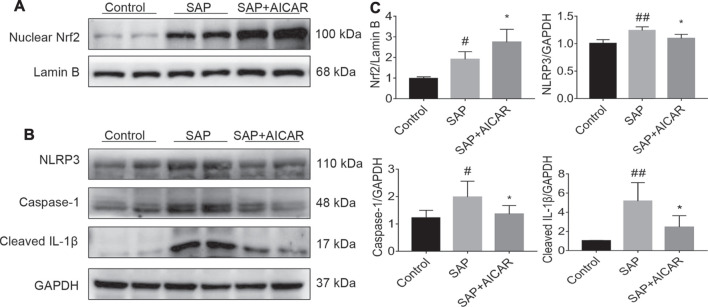
The effects of AICAR on the activation of the AMPK/Nrf2 signaling pathway and inhibition of NLRP3 inflammasome activation in the liver tissues of sodium taurocholate-induced SAP rats. 7–8 week-old wild-type male SD rats were randomly divided into three groups: the control group (control, n = 10) was treated with sham operation and injected with 0.9% normal saline; the severe acute pancreatitis (SAP) group (n = 10) was infused with 3% sodium taurocholate (0.1 ml/100 g); and the AMPK agonist (AICAR) group (n = 8) received intraperitoneal injection of AICAR (400 mg/kg) 1 h before the operation. **(A)** The levels of nuclear accumulation of Nrf2 in liver tissues of each group were detected by Western blot analysis. For the internal control, Lamin B was used. **(B)** The protein expression levels of NLRP3, caspase-1, and cleaved IL-1β in liver tissues were measured by Western blot using anti-NLRP3, anti-caspase-1 and anti-cleaved IL-1β antibodies. GAPDH was used as an internal control. **(C)** Relative band densities were quantified using VisionWorks imaging software, and the results are expressed in the histogram. Data are presented as the mean ± SD obtained from three independent experiments. ^#^ *p* < 0.05, ^##^ *p* < 0.01, ^###^ *p* < 0.001, ^####^ *p* < 0.0001 vs control group; ^*^ *p* < 0.05, **p < 0.01, ***p < 0.001, ****p < 0.0001 vs. SAP group.

Next, we focused on dissecting the deeper molecular mechanism by which AICAR inhibits oxidative stress and inflammation in the liver tissues of sodium taurocholate-induced SAP rats by activating AMPK phosphorylation. We performed Western blot to test the nuclear translocation of Nrf2 and the protein expression of NLRP3 as well as its downstream proteins caspase-1 and cleaved IL-1β in hepatic tissues of sodium taurocholate-induced SAP rats after treatment with AICAR. The nuclear translocation of Nrf2 was increased following sodium taurocholate treatment, whereas AICAR supplementation further promoted the nuclear accumulation of Nrf2 (Figures 4A,C). Moreover, sodium taurocholate treatment significantly increased the hepatic expression of NLRP3, caspase-1 and cleaved-IL-1β, while AICAR supplementation reversed this phenomenon (Figures 4B,C). These findings suggest that AICAR markedly alters the nuclear accumulation of Nrf2 and inhibits NLRP3 inflammasome activation in sodium taurocholate-induced PALI rats by activating AMPK phosphorylation. Thus, we speculate that Nrf2 and NLRP3 inflammasome pathway may mediate essential parts in the protective roles of AICAR against oxidative stress and inflammation in sodium taurocholate-induced PALI rats.

### Inhibition of AMPK Activation by Compound C Markedly Aggravates PALI in Sodium Taurocholate-Induced SAP Rats

**FIGURE 5:**
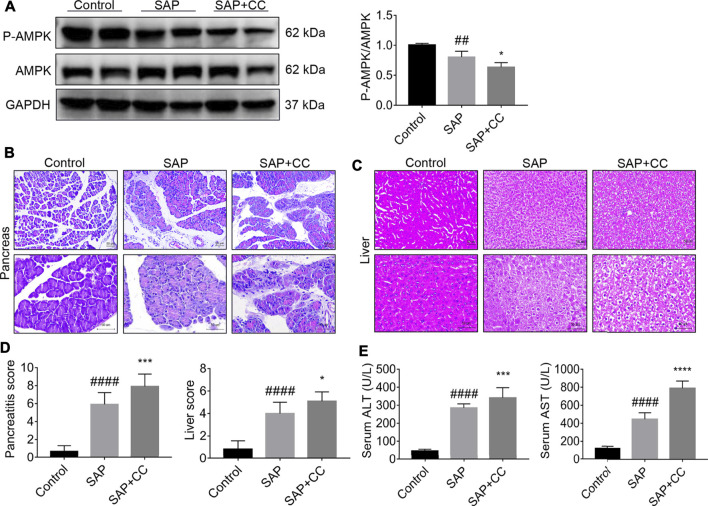
Inhibition of AMPK phosphorylation by Compound C markedly aggravates PALI in sodium taurocholate-induced SAP rats. Seven-to eight-week-old wild-type male SD rats were randomly divided into three groups: the control group (control, n = 10) was treated with a sham operation and injected with 0.9% normal saline; the severe acute pancreatitis (SAP) group (n = 10) was infused with 3% sodium taurocholate (0.1 ml/100 g); and the AMPK inhibitor (CC) group (n = 8) received intraperitoneal injection of CC (Compound C, 13.8 mg/kg) 1 h before the operation. **(A)** The protein expression levels of AMPK and phosphorylated AMPK (*p*-AMPK) in liver tissues were detected by Western blot using anti-AMPK and anti-*p*-AMPK antibodies. GAPDH was used as the internal reference control. Data are presented as mean ± SD of three independent experiments. **(B,C)** Histological images of H&E staining in pancreas and liver tissues (200x and 400x) under a light microscope (Bar = 50 μm). **(D)** Histological scores were obtained to evaluate the degree of pancreas and liver injury from H&E staining. **(E)** Serum ALT and AST concentrations were detected. Data are presented as the mean ± SD of three independent experiments. ^#^ *p* < 0.05, ^##^ *p* < 0.01, ^###^ *p* < 0.001, ^####^ *p* < 0.0001 vs control group; ^*^ *p* < 0.05, **p < 0.01, ***p < 0.001, ****p < 0.0001 vs. SAP group.

To further elucidate the role of AMPK in PALI, we treated rats with the AMPK inhibitor Compound C (CC, 13.8 mg/kg) by intraperitoneal injection to block the phosphorylation of AMPK in liver tissues, followed by sodium taurocholate infusion. Unsurprisingly, Western blot results showed that sodium taurocholate infusion significantly reduced the ratio of *p*-AMPK/AMPK in liver tissues. As expected, this ratio was further reduced by CC treatment, suggesting that CC treatment successfully inhibited AMPK phosphorylation levels in the liver tissues of SAP rats (Figure 5A). Interestingly, treatment with CC significantly exacerbated sodium taurocholate-induced pancreatic injury in rats, as evidenced by further increased acinar necrosis and inflammatory cell infiltration (Figure 5B). Evaluation of the pancreatitis score in pancreatic sections also revealed that CC treatment was accompanied by more severe pancreatic injury than SAP (Figure 5D). We also observed that administration of CC in rats augmented SAP-induced edema, necrosis and structural disorder in hepatic lobules with further increased liver injury scores compared with SAP rats (Figures 5C,D). In addition, the amplitude of SAP-induced elevation of serum levels of both ALT and AST, two markers of liver injury, in CC-treated rats was higher than that in the SAP groups (Figure 5E). The above results indicate that inhibition of AMPK phosphorylation by CC enhances sodium taurocholate-induced PALI in rats.

### AMPK Inhibition by Compound C Enhances Hepatic Oxidative Stress and NLRP3 Inflammasome Activation in Sodium Taurocholate-Induced PALI Rats

**FIGURE 6:**
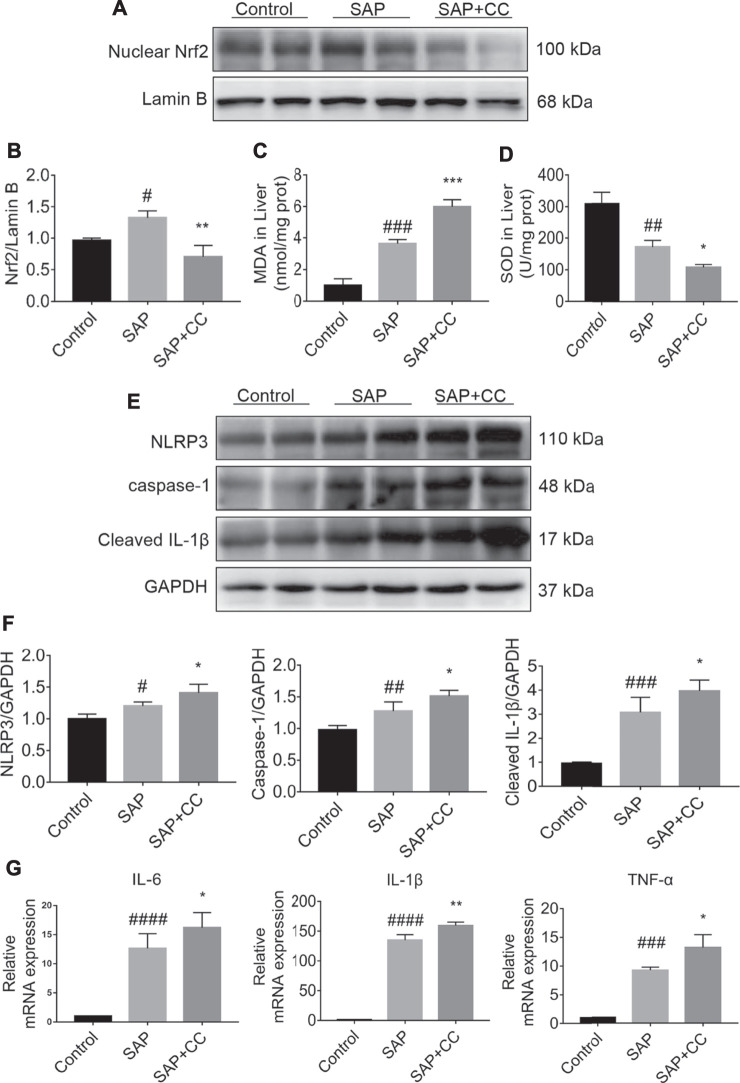
Inhibition of AMPK phosphorylation by Compound C enhances hepatic oxidative stress and inflammation levels in sodium taurocholate-induced SAP rats. Seven-to eight-week-old wild type male SD rats were randomly divided into three groups: the control group (control, n = 10) was treated with a sham operation and injected with 0.9% normal saline; the severe acute pancreatitis (SAP) group (n = 10) was infused with 3% sodium taurocholate (0.1 ml/100 g); and the AMPK inhibitor (CC) group (n = 8) received intraperitoneal injection of CC (Compound C, 13.8 mg/kg) 1 h before the operation. **(A)** The nuclear translocation levels of Nrf2 in liver tissues were determined by Western blot. For the internal control, Lamin B was used. **(C,D)** The contents of MDA and SOD in liver tissues were determined. **(E)** The protein expression of NLRP3, caspase-1, and cleaved IL-1β was detected by Western blot. GAPDH was used as the internal reference control. **(B,F)** The relative ratios of Nrf2, NLRP3, caspase-1, and cleaved IL-1β were quantified using VisionWorks imaging software. **(G)** The mRNA levels of IL-6, IL-1β, and TNF-α in liver tissues were measured by RT-qPCR. Data are presented as the mean ± SD of three independent experiments. ^#^ *p* < 0.05, ^##^ *p* < 0.01, ^###^ *p* < 0.001, ^####^ *p* < 0.0001 vs control group; ^*^ *p* < 0.05, **p < 0.01, ***p < 0.001, ****p < 0.0001 vs. SAP group.

We next explored whether inhibition of AMPK activation by CC could promote hepatic oxidative stress and inflammation levels in sodium taurocholate-induced SAP rats. We found that the increase in the nuclear translocation of Nrf2 in the hepatic tissues of SAP rats was markedly reduced after CC treatment (Figures 6A,B). We further measured MDA and SOD levels, which are indicators of oxidative damage, in the liver tissues of each group. The levels of hepatic MDA in rats treated with CC were significantly augmented compared to those in the SAP groups (Figure 6C). Alternatively, treatment with CC had a reduced effect on the magnitude of sodium taurocholate-induced decline in hepatic SOD levels (Figure 6D). Moreover, these changes in CC-treated SAP rats were accompanied by significantly increased hepatic expression of NLRP3, caspase-1 and cleaved IL-1β compared with SAP rats (Figures 6E,F), which results in a certain type of inflammatory response-related cell death called pyroptosis (Guo et al., 2021). Thus, we next compared the levels of IL-6, IL-1β and TNF-α in the liver tissues of SAP rats by q-PCR analysis with or without CC treatment. Consistent with our previous findings, the expression of these inflammatory cytokines was upregulated in the SAP groups, whereas CC treatment led to a further increase in the mRNA abundance of the aforementioned inflammatory genes (Figure 6G). Ultimately, our data suggest that inhibition of AMPK phosphorylation by CC aggravates PALI in sodium taurocholate-induced SAP rats, likely by repressing Nrf2-mediated antioxidant stress and anti-inflammatory roles.

### Nrf2 Knockout Weakens the Protective Effects of AICAR on PALI in L-Arginine-Induced SAP Mice

**FIGURE 7:**
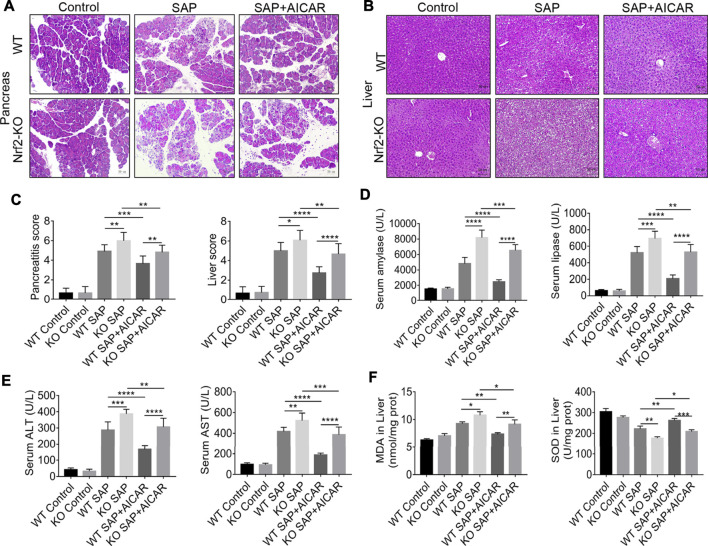
Nrf2 knockout attenuates the protective effects of AICAR on PALI and the inhibitory effects of hepatic oxidative stress in L-arginine-induced SAP mice. Male wild-type mice or Nrf2^−/−^ mice were divided into three groups: the control group (control, n = 6) was intraperitoneally injected with 0.9% normal saline; the SAP group (n = 6) was intraperitoneally injected with 8% L-arginine hydrochloride (pH = 7.0, 4 g/kg) twice at an interval of 1 h; and the AMPK agonist (AICAR, n = 6) group was intraperitoneally injected with AICAR (400 mg/kg) twice: 1 h before and 24 h after L-arginine hydrochloride administration. **(A,B)** Representative pathological images of the pancreas and liver (200x) under a light microscope (H&E staining, Bar = 50 μm). **(C)** Histological scores were obtained to evaluate the degree of pancreas and liver injury from H&E staining. **(D)** The concentrations of serum amylase and lipase were detected. **(E)** The levels of serum ALT and AST were measured to evaluate liver function. **(F)** Concentrations of MDA and SOD in liver tissues from different groups. Data are presented as the mean ± SD of three independent experiments. Data are presented as mean ± SD of three independent experiments. ^*^ *p* < 0.05, **p < 0.01, ***p < 0.001, ****p < 0.0001.

Since the aforementioned data indicate that the anti-inflammatory and antioxidant stress effects of AICAR may be related to the induction of Nrf2 nuclear translocation, we further investigated whether Nrf2 deficiency would affect the protective effects of AICAR against PALI in L-arginine-induced SAP mice. L-arginine-induced elevations in serum levels of pancreas injury enzymes (amylase and lipase) and the pathological changes as well as pancreatitis scores analyzed in H&E-stained pancreas sections in Nrf2 knockout (KO) mice were higher than those in WT SAP mice (Figures 7A–D). Meanwhile, the beneficial effects of AICAR against L-arginine-induced pancreatic injury reflected by the above indicators were significantly attenuated in Nrf2 KO mice compared with WT littermates (Figures 7A,C). Likewise, the pathological changes in liver sections of the Nrf2 KO SAP mice were more severe than those in the WT SAP groups by H&E staining (Figure 7B). Additionally, treatment of WT SAP mice with AICAR markedly reduced these negative pathological changes (Figure 7B); however, these protective effects mediated by AICAR were weakened in Nrf2 KO mice (Figure 7B). The liver injury scores, consistent with the pathological appearance in each group, further confirmed our findings (Figure 7C). Moreover, the serum levels of ALT and AST in both WT and Nrf2 KO mice were augmented after L-arginine administration. Notably, the levels of these two markers indicated that liver injury in Nrf2 KO mice was higher than that in WT mice (Figure 7E). Alternatively, AICAR treatment markedly attenuated the L-arginine-induced elevation in the serum levels of ALT and AST in WT SAP mice, while these phenomena were significantly inhibited in Nrf2 KO mice (Figure 7E). Therefore, these results indicate that Nrf2 plays an important role in the protective effects of AICAR against L-arginine-induced PALI in mice.

### Nrf2 Deficiency Impairs the Inhibitory Effects of AICAR Against PALI-Related Hepatic Oxidative Stress and NLRP3 Inflammasome Activation in L-Arginine-Induced SAP Mice

To further clarify whether the beneficial effect of AICAR on antioxidant stress was Nrf2 dependent, we measured MDA and SOD levels in the liver tissues of L-arginine-treated Nrf2 KO mice and WT littermates with or without AICAR supplementation. After L-arginine treatment, the production of MDA in liver tissues was conspicuously increased, and the levels of SOD were significantly decreased in Nrf2 KO mice compared to WT mice. Additionally, administration of AICAR was associated with markedly reduced MDA levels, and the concentrations of SOD were significantly augmented compared to those in the WT SAP group. However, these beneficial antioxidant effects of AICAR were largely blocked in Nrf2 KO mice compared to WT littermates (Figure 7F). These observations indicate that the antioxidant ability of AICAR in the liver tissues of L-arginine-induced PALI mice was partially dependent on Nrf2.

**FIGURE 8:**
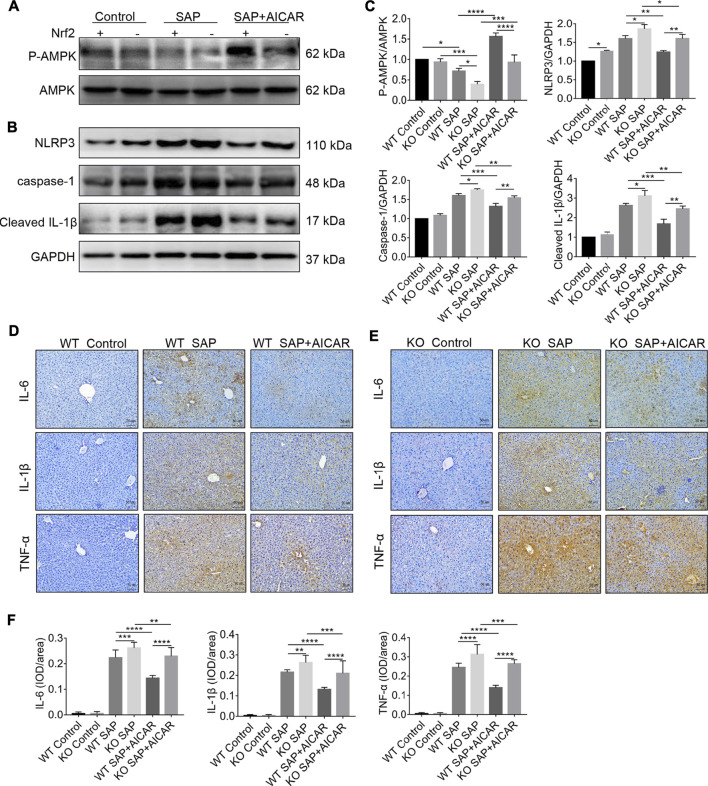
Nrf2 deficiency impairs the inhibitory effects of AICAR against hepatic NLRP3 inflammasome activation in L-arginine-induced SAP mice. Male wild type mice or Nrf2^−/−^ mice were divided into three groups: the control group (control, n = 6) was intraperitoneally injected with 0.9% normal saline; the SAP group (n = 6) was intraperitoneally injected with 8% L-arginine hydrochloride (pH = 7.0, 4 g/kg) twice at an interval of 1 h; and the AMPK agonist (AICAR, n = 6) group wasintraperitoneally injected with AICAR (400 mg/kg) twice: 1 h before and 24 h after L-arginine hydrochloride administration. **(A–C)** The protein expression levels of phosphorylated AMPK, total AMPK, NLRP3, caspase-1, and cleaved IL-1β were measured by Western blot, and the relative ratios of *p*-AMPK/AMPK and band densities of Nrf2, NLRP3, caspase-1, and cleaved IL-1β were quantified using VisionWorks imaging software. GAPDH was used as an internal reference. **(D–F)** The protein expression of IL-6, IL-1β and TNF-α in liver sections was detected by immunohistochemistry (original magnification ×200, Bar = 50 μm). Image-Pro Plus 6.0 software was used for statistical analysis. Data are presented as the mean ± SD of three independent experiments. ^*^ *p* < 0.05, **p < 0.01, ***p < 0.001, ****p < 0.0001.

We next determined whether induction of Nrf2 is required for the inhibition of the NLRP3 inflammasome-associated inflammatory response in the liver tissues of L-arginine-induced PALI mice. Thus, we performed Western blot to compare the hepatic expression levels of NLRP3, caspase-1 and cleaved-IL-1β between L-arginine-treated Nrf2 KO mice and WT littermates with or without AICAR administration. The results showed that the hepatic expression of NLRP3, caspase-1 and cleaved-IL-1β in Nrf2 KO mice was higher than that in WT littermates after L-arginine treatment (Figures 8A–C). Furthermore, replenishment of AICAR profoundly decreased the expression of these indicators in the liver tissues of WT littermates compared with the WT SAP group; however, these observed trends were significantly reversed in Nrf2 KO mice (Figures 8B,C). Thus, our findings suggest that Nrf2 deficiency impairs the inhibitory effect of AICAR against NLRP3 inflammasome activation in L-arginine-induced PALI mice. To further elucidate the role of Nrf2 in the anti-inflammatory effects of AICAR, we detected the expression levels of several inflammatory factors, including IL-6, IL-1β and TNF-α, in each group of Nrf2 KO mice and WT littermates. Immunohistochemistry analysis showed that L-arginine induced the expression of these inflammatory factors in the liver tissues of both Nrf2 KO mice and WT littermates, resulting in a further increase in their expression (Figures 8D–F). Moreover, replenishment of AICAR decreased L-arginine-induced hepatic inflammatory factor accumulation in WT littermates, while these anti-inflammatory effects of AICAR were markedly reversed in Nrf2 KO mice compared with WT controls (Figures 8D–F). Taken together, our results suggest that Nrf2 deficiency largely eliminates the negative regulation of AICAR on the NLRP3 inflammasome activation associated inflammatory response in the liver tissues of L-arginine-induced PALI mice.

## Discussion

The central goal of this study was to investigate the pharmacological activation of AMPK by AICAR as a therapeutic strategy for the treatment of PALI. Our findings demonstrated that AICAR activates AMPK, which leads to Nrf2-mediated antioxidant stress and inhibition of NLRP3-related inflammation, and thus improving PALI. This study indicated that AMPK exerted an essential role in the pathological processes of PALI and presented the first evidence that pharmacological activation of AMPK by AICAR ameliorates PALI, suggesting that AICAR may be a promising therapeutic agent for the treatment of PALI.

In the current study, we demonstrated that both 1 day after sodium taurocholate infusion in rats and 2 days after intraperitoneal injection of L-arginine in mice successfully induced rodent models of PALI, as evidenced by marked elevation in the plasma levels of amylase, lipase, ALT and AST as well as severe pathological injury of the pancreas and liver in the two experimental SAP models (Figures 1B–G, Figures 7A–F). This study provided novel evidence that SAP caused a drastic reduction in hepatic expression levels of phosphorylated AMPK in sodium taurocholate- and L-arginine-induced rodent SAP models, suggesting that AMPK may be involved in the pathogenesis of PALI (Figure 1A, Figures 8A,C). Herein, we obtained two lines of evidence to demonstrate our hypothesis. On the one hand, administration of the direct AMPK activator AICAR led to a dramatic elevation in hepatic expression levels of phosphorylated AMPK and prevented PALI in sodium taurocholate- and L-arginine-induced rodent SAP models (Figures 1A–G, Figures 7A–E, Figures 8A,C). On the other hand, inhibiting the activity of AMPK in liver tissues by using the AMPK inhibitor Compound C profoundly exacerbated PALI in sodium taurocholate-induced SAP rats (Figures 5A–E). Although previous studies have shown that compound C may play roles independent of AMPK inhibition, such as anti-glioma and inhibition of Bone morphogenetic protein (BMP) pathway, it is still the only available cell permeability AMPK antagonist (Yu et al., 2008; Liu et al., 2014). Similar to our findings, AMPK activation by AICAR has been shown to protect the kidney from overt damage in a rat model of kidney ischemia-reperfusion injury (Lempiäinen et al., 2012). In addition, a Compound C-induced reduction in myocardial AMPK activity impaired cardiac function and aggravated oxidative stress in cardiomyocytes in DOX-induced acute cardiotoxicity mice (Liu et al., 2018).

In the pathogenesis of PALI, the uncontrollable hepatic inflammatory response and elevated oxidative stress triggered by SAP will continue to damage hepatocytes, which are central mediators for accelerating the development of PALI (Yang et al., 2003; Moniaux et al., 2011). According to previous reports, AMPK activation is involved in the inhibition of the IL-6-stimulated inflammatory response in human liver cells and mediates the antioxidant stress ability of fibroblast growth factor (FGF) one in a mouse model of nonalcoholic fatty liver disease (Nerstedt et al., 2013; Liu et al., 2020). Therefore, we explored the hypothesis that the molecular basis of AICAR in improving PALI is attributed to its anti-inflammatory capability and reduction of hepatic oxidative stress by AMPK activation. Our findings demonstrated that AMPK was required for the regulation of hepatic inflammation and oxidative stress in sodium taurocholate-induced PALI rats and that AICAR ameliorated PALI by AMPK activation in this rat model, which was associated with its anti-inflammatory and antioxidative actions.

Next, we focused on dissecting the molecular mechanism to explain how AMPK activation by AICAR reduced hepatic inflammation and oxidative stress in PALI. In response to inflammatory reaction, Nrf2 is released from Keap1 and translocates to the nucleus, thus activating the endogenous antioxidant defense system by enhancing the transcription of a variety of antioxidant enzymes, such as HO-1 and NQO-1, presenting a superior effect in oxygen reduction balance. In particular, oxidative stress and inflammation are closely connected, and increased levels of proinflammatory cytokines induce oxidative stress. In turn, oxidative stress overactivation further exacerbates the inflammatory response (Dandekar et al., 2015; Antonucci et al., 2017). Furthermore, studies have also shown that the activation of Nrf2 can inhibit inflammation-mediated injury *via* its antioxidant cascade (Joo Choi et al., 2014; Saha et al., 2020). Notably, several recent studies have demonstrated that Nrf2 negatively regulates the activity of the NLRP3 inflammasome, which is a central regulator of inflammation required for PALI (Hou et al., 2018; Chen et al., 2019). Upon stimulation, NLRP3 collects ASC protein and converts procaspase-1 into active caspase-1, which cleaves pro-IL-β or pro-IL-18 into mature forms, ensuing and promoting hepatic inflammatory injury (Mridha et al., 2017). However, the crosstalk between Nrf2 and the NLRP3 inflammasome pathway in PALI remains to be determined. In addition, previous reports found that AMPK also inhibits NLRP3 inflammasome activation in a mouse model of diabetic cardiomyopathy and acute pancreatitis (Chen et al., 2020; Yang et al., 2020). In fact, AMPK phosphorylates Nrf2 at serine 374, 408, and 433, moves Nrf2 from the cytoplasm to the nucleus, and binds to the antioxidant response element (ARE) gene to exert its antioxidant effect (Matzinger et al., 2020). In the current study, rats treated with AMPK agonists (AICAR) and inhibitors (CC) demonstrated that AMPK plays an important role in activating Nrf2 signaling and inhibiting NLRP3 inflammasome pathways in PALI and that hepatic Nrf2 may act as a key mediator of AICAR protection against PALI-associated oxidative stress and NLRP3 inflammasome activation. Therefore, we speculate that the direct activation of AMPK by AICAR exerts its antioxidant and anti-inflammatory roles to prevent PALI through Nrf2-mediated antioxidant effects and inhibition of the NLRP3 inflammasome pathway.

To verify our hypothesis, we used Nrf2 KO mice and WT mice to conduct a comparative study in an L-arginine-induced PALI model with or without AICAR treatment. We found that knockout of Nrf2 limited the ability of AICAR to reduce the severity of PALI in mice (Figures 7A–E). Most importantly, Nrf2 gene deletion markedly weakened the protective effects of AICAR to prevent SAP-induced oxidative stress and NLRP3 inflammasome activation in the liver tissues of L-arginine-induced PALI mice (Figure 7F, Figures 8B,C). However, AICAR still had a slight protective effect on PALI in Nrf2 KO mice. AMPK can regulate a variety of physiological and pathological effects through multiple pathways to affect cell metabolism and survival (Carling, 2017; Ramirez Reyes et al., 2021). This result may be attributed to the activation of AMPK by AICAR leading to other pathway modulations independent of Nrf2 signaling in PALI and resulting in other biological effects, such as AMPK-related mitochondrial homeostasis, ferroptosis and necroptosis, which are worthy of further exploration in our future research (Herzig et al., 2018; Song et al., 2018; Lee et al., 2020). Nonetheless, our findings indicate that activation of Nrf2 by AICAR mediates important roles in ameliorating hepatic oxidative stress and inhibiting NLRP3 inflammasome pathway activation in PALI mice regardless of whether Nrf2 is the master pathway.

Despite our novel findings, the current study has some limitations. First, our observations are only based on two rodent PALI models. AICAR has been used clinically for myocardial protection in coronary artery bypass grafting and myocardial ischemic injury (Rao et al., 2016). We should further determine the role of AICAR in PALI in humanoid large animals and in clinical studies. Second, our results demonstrated that AICAR protects against PALI partially through Nrf2-mediated antioxidant effects and inhibition of NLRP3 inflammasome activation, whether AICAR plays these positive roles mainly depends on reducing the activation of inflammatory cells, such as macrophages and neutrophils, exerting a protective effect on hepatocytes or inducing other hepatoprotective factors requires further investigation. Third, although AICAR is a widely used AMPK agonist, it also plays important AMPK-independent effects including regulating gluconeogenesis and oxidative phosphorylation (OXPHOS). Therefore, in addition to its inhibitory effects on inflammation and oxidative stress by activating ampk, AICAR may play protective roles of PALI through these AMPK-independent pathways which need to be further explored (Višnjić et al., 2021). Fourth, this study predominantly focuses on the AMPK/Nrf2 signaling pathway. However, there are also many other important regulatory pathways involved in the course of PALI such as Toll like receptor 4 (TLR4) signaling pathway, transforming growth factor β1 (TGF-β1) and p38 mitogen-activated protein kinase (MAPK) signaling pathway (TGF-β1-p38 MAPK), which need to be investigated in our future study (Wang et al., 2018). Fifth, given the powerful therapeutic effect of AICAR in PALI, the potential effect of AICAR in other organ dysfunctions including respiratory, renal, and cardiovascular of SAP needs to be further explored.

**FIGURE 9:**
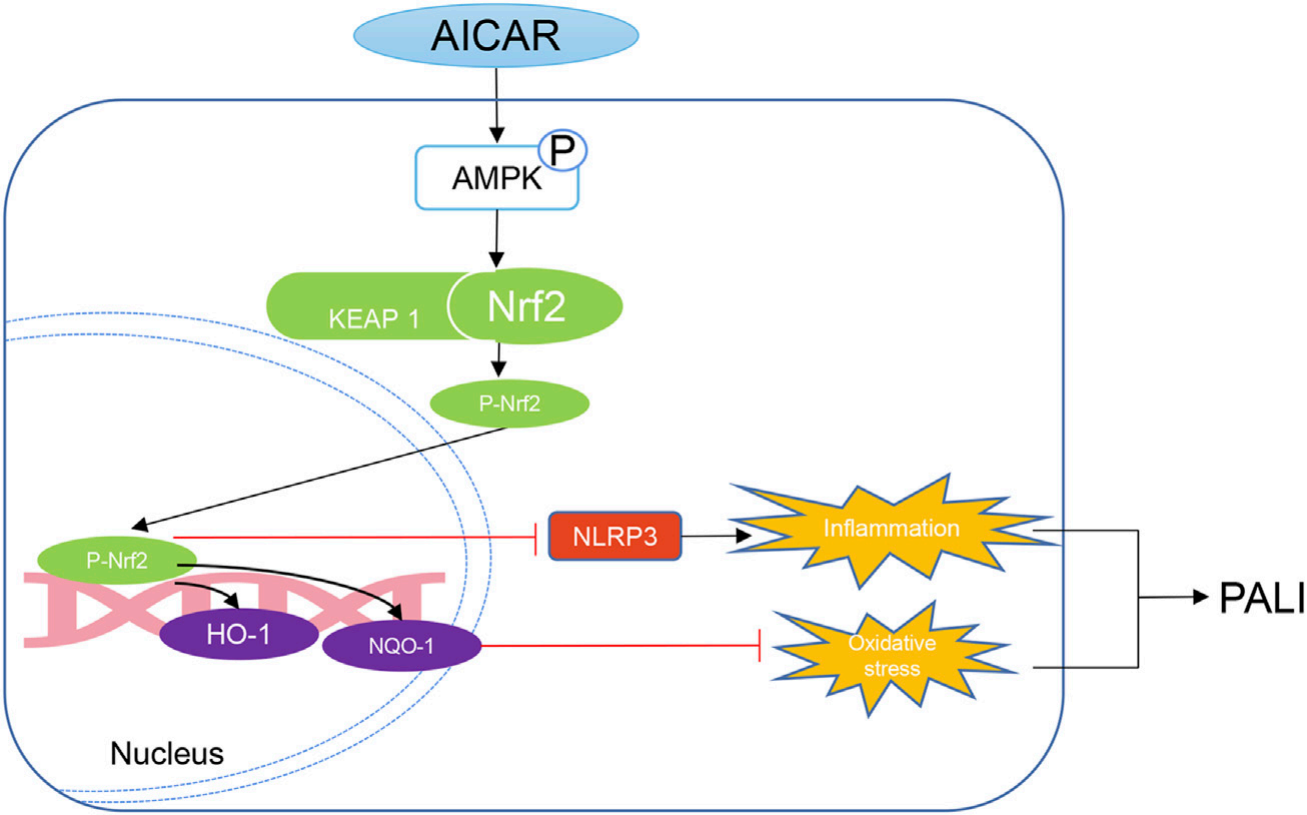
Mechanistic illustration of the basis of AICAR protection against pancreatitis-associated liver injury (PALI). AICAR inhibits hepatic oxidative stress and inflammation by promoting AMPK phosphorylation partially *via* Nrf2-mediated antioxidant effects and inhibition of NLRP3 inflammasome activation, resulting in protection against PALI in sodium taurocholate- and L-arginine-induced SAP models.

In summary, our data indicate that AICAR, a direct AMPK activator, exhibits significant therapeutic effects against PALI in sodium taurocholate- and L-arginine-induced rodent models by promoting AMPK phosphorylation by effectively inhibiting hepatic oxidative stress and inflammation. Thus, as a cell permeable nucleoside, AICAR has high therapeutic value for the treatment of PALI. Importantly, this study provides new insight into the mechanisms underlying the improvement of hepatic oxidative stress and inflammation in PALI by AICAR. AMPK activation promotes the nuclear accumulation of Nrf2, which partially mediates antioxidant effects and inhibits NLRP3 inflammasome activation and thus is important for AICAR protection against PALI (Figure 9). We conclude that because AICAR is already used in the clinic, the development of novel therapies using AICAR to promote AMPK phosphorylation is promising for future medical interventions of PALI.

## Data Availability

The original contributions presented in the study are included in the article/Supplementary Material, further inquiries can be directed to the corresponding authors.
