# Supplementary material for: AICAR, an AMP-Activated Protein Kinase Activator, Ameliorates Acute Pancreatitis-Associated Liver Injury Partially Through Nrf2-Mediated Antioxidant Effects and Inhibition of NLRP3 Inflammasome Activation
Source: Front Pharmacol. 2021 Aug 31;12:724514. doi: 10.3389/fphar.2021.724514 (PMC8438129; doi:10.3389/fphar.2021.724514)
Supplement: Supplementary file 1 [file Table1.pdf]

Gene	Forward primer (5′–3′)	Reverse primer (5′–3′)
GAPDH	ACCACAGTCCATGCCATCAC	TCCACCACCCTGTTGCTGTA
IL-6	GAGTTGTGCAATGGCAATTC	ACTCCAGAAGACCAGAGCAG
IL-1β	AGGCTGACAGACCCCAAAAG	CTCCACGGGCAAGACATAGG
TNF-α	AAATGGGCTCCCTCTCATCAGTTC	TCTGCTTGGTGGTTTGCTACGAC
